# A new method for birch tar making with materials available in the Stone Age

**DOI:** 10.1038/s41598-021-04161-3

**Published:** 2022-01-10

**Authors:** Tabea J. Koch, Patrick Schmidt

**Affiliations:** 1 Department of Early Prehistory and Quaternary Ecology, Eberhard Karls University of Tübingen, Tübingen, Germany; 2 Applied Mineralogy, Department of Geosciences, Eberhard Karls University of Tübingen, Tübingen, Germany

**Keywords:** Archaeology, Cultural evolution

## Abstract

The use of birch tar can be traced back to the European Middle Palaeolithic and is relevant for our understanding of the technical skills and cognitive abilities of Neanderthals. Due to the lack of archaeological evidence, it remains unknown what techniques were used for birch tar making. Efficiency was recently used as a proxy to determine the method most likely used in the Middle Palaeolithic. Todtenhaupt et al. have proposed a technique employing a groove-like structure that is comparable with the recently presented condensation method. The groove method resulted in higher tar yields compared to other experimental aceramic production processes. However, the implications for Palaeolithic tar making remain unclear because some of the materials used in the experiment were not available then (polished granite slabs). To approach this problem, we replicated the groove with river cobbles and, in a second experiment with flint fragments, to evaluate whether similar results can be obtained. We were successful in producing birch tar in multiple runs with the cobble- and flint-grooves, which, in addition, proved to be more efficient than the condensation method in terms of tar yield per bark input. Our experimental study provides an additional possibility to make prehistoric birch tar.

## Introduction

Birch tar is known from the European Middle Palaeolithic either as lumps or hafted to stone tools. All five known pieces can be associated with Neanderthals. They were found at Campitello Quarry, Italy (< 200 ka)^1^, Königsaue, Germany (~ 80 to 43 ka)^2–4^; and Zandmotor, The Netherlands (~ 50 ka)^5^. A large number of lithic artefacts and some cobbles covered by a blackish residue proposed to be birch tar were discovered in the Inde Valley, Germany, and are associated with the Micoquien (~ 120 ka)^6^. However, no gas-chromatographic analysis has been conducted to confirm that the Inden-Altendorf findings are birch tar. The production techniques used by Neanderthals to make birch tar are still unknown, despite the large corpus of, mainly experimental, studies that have attempted to shed light on the question^7–15^. The investigated methods can be divided into autothermic and allothermic birch tar making techniques^10,16^. Autothermic means that methods are based on directly lighting the birch bark itself. An example is the condensation method where bark is burned beside river cobbles^14^. Allothermic means that the bark is heated by a source other than itself. Techniques based on allothermic processes include kiln-like structures, such as the raised structure where bark is sealed in earthen structures that are heated from the outside^8,9,12,13^. Other allothermic processes include the ash mound, where bark is heated in a mound of embers and ashes, and the pit roll technique where bark is heated in earthen pits that are heated from above^9,11,17^. Some of these techniques may be understood to approximate the better-known single pot technique (although this technique normally uses ceramic containers, see for example:^18,19^). In a previous study^20^, the temperature range at which birch tar forms in such single-pot conditions was found to lie between 350 and 400 °C. More complex techniques were found to allow for higher tar yield but require more time and effort^9^. Some authors^21^ draw conclusions on the cognitive abilities of Neanderthals based on the degree of complexity of different methods. However, without artefacts directly associated with the production process of birch tar making, we cannot determine the exact method employed by Neanderthals. Several parameters can be used to propose the probability that one or another method was used. This is, for example, the efficiency of different methods, that can be investigated experimentally^5,7,9^. In this regard, the raised structure is described as highly efficient^9^. This has led some authors^5^ to propose that it was more likely than other less efficient methods, such as the condensation method^14,22^. Other arguments for or against specific methods are the performance of tars made with them (see for example:^14^ or their technical complexity (see for example:^23^).

The condensation method consists of using a cobble with a flat tilted surface, so to create an overhang to condense tar on the surface. A roll of birch bark is lit beside the overhanging surface. A method that, in some regard, is similar was proposed by Todtenhaupt et al.^15^ who used a groove structure lined with flat and polished granite slabs. This groove was placed on a rotatable platform (and turned in the direction of the wind) with a slope of 10° at the bottom of the groove. The groove had a width of 6.5 cm, a height of 5 cm, and a length of ~ 20 cm. Strips of birch bark were placed in this groove. Another granite panel was used to cover the structure. The bark was lit and burned for 23–30 min^15^, leading to tar condensation on the granite panels. The optimal quantity of bark in such a structure was described as 50 g of bark cut into 15 cm × 2.5 cm strips. To regulate combustion, the openings on each side of the groove could be opened and the cover plate was moved to allow air circulation^15^. This groove allowed to produce ~ 1 cm^3^ of tar that could be scraped off the granite slabs^15^. Thus, it appears to be more efficient than the condensation method by a factor greater than two^7^. The problem with this method for interpreting Neanderthal birch tar production is that polished granite slabs were not available in the Middle Palaeolithic. In order to evaluate this method, a replication with naturally occurring materials is essential to understand possible Prehistoric birch tar making techniques. In a similar approach described by Meijer and Pomstra^11^, it may be understood that they experimented with a cobble-lined pit in which birch bark is burned (autothermic). Unfortunately, they do not report whether their experiment was successful or not.

In this study, we replicate Todtenhaupt et al.’s^15^ groove, attempting to find a way of construction using materials that were available in the Palaeolithic. We compare the efficiency of this structure with the conventional condensation method. The aim is to evaluate whether the method (we call it the cobble-groove condensation method) successfully produces birch tar and to investigate its efficiency. For this, we directly compare two experimental series, one conducted with the conventional condensation method, the other with the cobble-groove. We also built a groove structure with fragments of flint instead of cobbles, to evaluate whether this type of construction can be considered as a potential method. If the cobble-groove technique produces more tar per time and bark than the condensation method, it may be argued that this method is a possible evolution of the simpler condensation method. Another aim is to assess whether the amount of tar produced using this method is comparable to the artefacts recovered at the Neanderthal sites that have yielded birch tar.

## Materials and methods

### Materials

For the condensation method, 3 large cobbles with at least one flat side and 132 g of dead birch bark (Betula pendula) were used. Nineteen cobbles with at least one flat surface were collected to provide options for the construction of the structure for the cobble-groove. All cobbles were river-rounded and presented low surface relief to the point that no surface roughness could be felt to the touch. Cobbles were either limestone, fine-grained sandstone or extrusive volcanics (not further specified). Twenty-six fragments of flint with at least one flat surface were knapped for the construction of a flint-groove. Sediment was taken from the surrounding forest area. For the groove condensation methods, a total of 358 g of bark was collected from approximately 6 dead birch trees lying on the ground of a birch forest. We only collected well-preserved bark from the trunk portions of the trees using knapped flint tools. This represented 30 min of collection time for two persons. However, the amount of time spent on collecting the bark is a general estimate based on the environmental conditions encountered in this study (see photos in the Supplementary Information).

### Experimental setup

**Figure 1:**
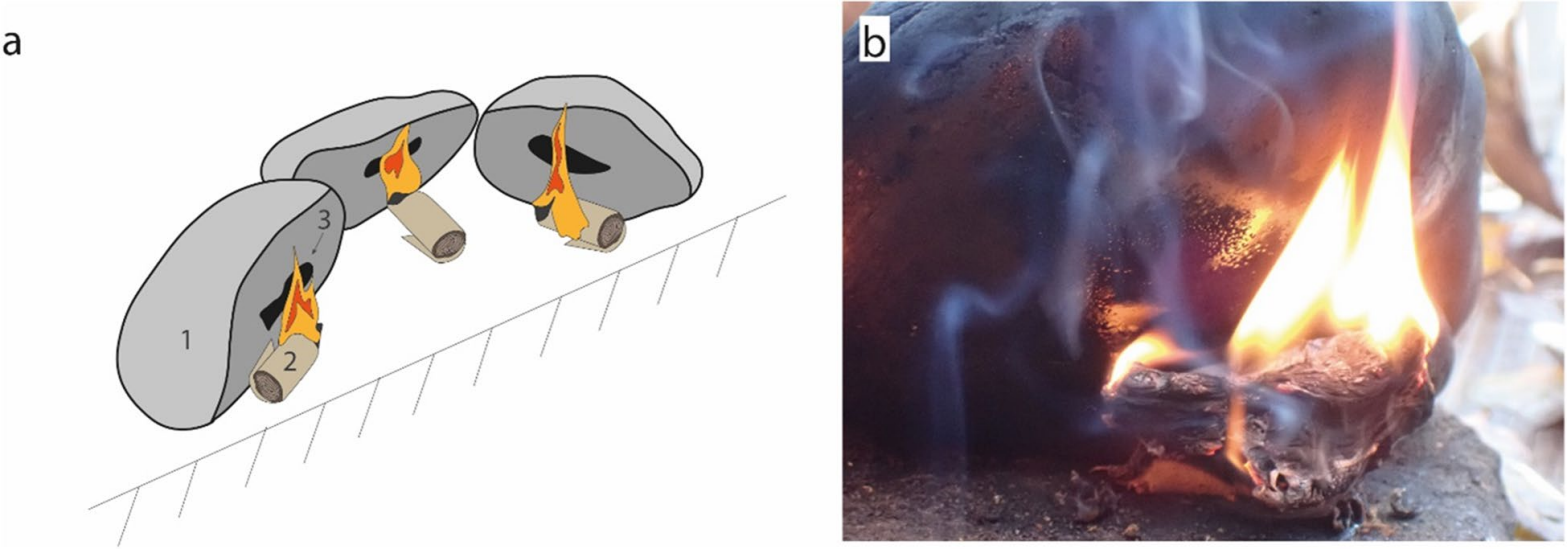
Experimental birch tar production with the condensation method. (**a**) Experimental set up of three large cobbles (1) with their flat surface slightly tilted to create an overhang. The bark fragments or rolls (2) were lighted and placed beneath the cobbles on which the condensed birch tar (3) is deposited. (**b**) Close up image of one cobble with a partially burnt bark roll and a shiny dark layer of tar accumulating on the cobble surface.

The condensation method was conducted following the protocol described in Schmidt et al.^14,22^. We used three large cobbles simultaneously that were placed on a flat surface with their flat side tilted to create an overhang (Fig. 1a). The cobbles were placed with that overhang facing towards the operator of the experiment (TJK). The experiment started by lighting rolled up bark pieces that were placed under the overhang of the cobbles (Fig. 1b). After 2–3 bark fragments burned out beside one cobble, the cobble was lifted and birch tar that had condensed beside the flame was scraped off with a stone tool (knapped flint flake). This procedure was repeated throughout the experiment. The condensation method was performed twice for 30 min using 80 g and 52 g of bark (two videos, showing the exact same protocol, are shown in^7^).

**Figure 2:**
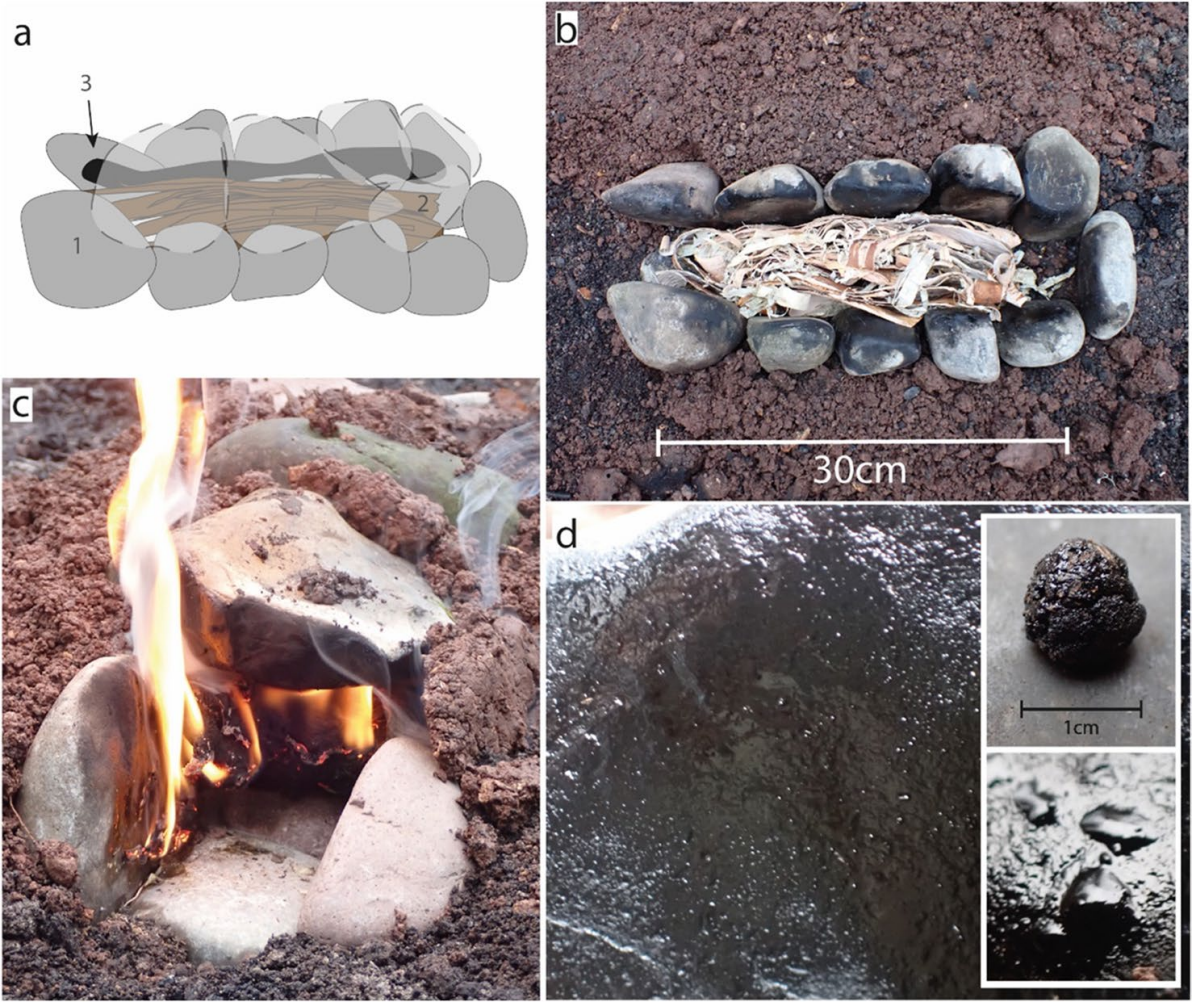
Experimental birch tar production with the cobble-groove condensation method. (**a**) Experimental set up of cobble-groove (1) showing the wall cobbles in dark grey, the ceiling cobbles with a dashed outline; the three bottom cobbles are hidden underneath the bark strips (2). The birch tar (3) is condensed onto the wall and ceiling cobbles. (**b**) Photo taken during the fourth experiment with clearly visible dark spots on the cobbles. (**c**) Frontal view of the upper opening of the structure during the burning procedure showing the bottom cobbles and the sediment chunks filling the gaps between the cobbles. (**d**) Close-up photo of condensed tar on a cobble, photo of a 0.27 g-piece of tar produced in 22 min, and a close-up photo of tar droplets.

For the cobble-groove condensation method, we followed the descriptions in Todtenhaupt et al.^15^, modifying it in a way that only materials available in the Palaeolithic were used (Fig. 2a). Instead of polished granite slabs, we used naturally rounded cobbles to build a groove-like structure (Fig. 2b). We dug a 40 cm long groove with a bottom inclined at ~ 10° (however, the last experiment, CGCM (6) in Table 1, was conducted with a flat groove that had no inclination). We paved the groove with three cobbles to create a flat surface with a width of ~ 6.5 cm (visible in Fig. 2c. During experiments 1 and 2, we placed four cobbles on each side of the groove. This allowed for an overall depth of the groove of 4–5 cm. We placed 2-3 cm wide strips of birch bark in the structure (50 g in experiment 1 and 51 g in experiment 2). Five cobbles were placed on top of the structure to cover it (flat surfaces of the cobbles were directed inwards of this structure on all sides). The gaps between cobbles were filled with sediment for all experiments except experiment 2. The exception was made to test whether more oxygen supply facilitates burning and/or impedes tar formation. It took 12 min to set up the experiments (digging the hole: 5 min; placing the stones: 2 min; placing the bark: 2½ min; placing the top stones: ½ min; filling the holes with sediment: 2 min). Airflow was regulated by a removable cobble at the lower end of the inclined groove. Experiments 3–6 were based on a slightly modified structure. To allow for better air circulation, the number of cobbles on each side was increased to five while positioning the flat surface of the rocks in an upright vertical position that increased the depth of the structure. This added 2 cm to the height of the walls (creating a ~ 5 to 7 cm deep groove). Experiment 3 was conducted with 30 g of birch bark, to test a looser packing of the bark strips. In experiments 4–6, we used 50 g of birch bark. To better understand the temperature conditions within such a cobble-groove, a thermocouple was inserted into the structure during experiment 6 (reaching into the structure between two wall cobbles and above the bark strips). However, we do not expect that these temperature measurements are meaningful, as temperature is only measured at one specific location and will most likely fluctuate depending on the position of the flames in the groove. In each experimental sequence, we lit the birch bark strips at the open end on the upper part of the inclined cobble-groove. When the flames threatened to go out, we blew (using our mouth, not a fan) into the open end of the structure and, when necessary, the cobble at the lower end was lifted to allow supplementary airflow. This did require that we observed the structure and checked from time to time that the fire would not go out. After combustion of the bark strips, which took on average 15–20 min, the sediment was removed. The top and wall cobbles were lifted, and birch tar was scraped off using stone tools. The bottom cobbles were not scraped because no tar deposited onto them. After each experiment, the cobbles were left to cool to room temperature before starting the next experiment (a video showing the construction and one run of the cobble-groove condensation method, experiment CGCM (6) of Table 1, is shown in Supplementary Video S1).

**Table 1:** Results of eight birch tar production experiments with the condensation method (CM), the cobble-groove condensation method (CGCM) and the flint-groove condensation method (FGCM).

Method (#)	Bark	Used bark (g)	Time (min)	Tar yield (g)	Tar/h (g)	Tar/ 100 g bark (g)	Comments
CM (1)	Dead bark	80	30	0.27	0.54	0.34	Three cobbles in use simultaneously
CM (2)	Dead bark	52	30	0.28	0.56	0.54	Three cobbles in use simultaneously
CGCM (1)	Dead bark	50	27	0.16	0.36	0.32	17 cobbles, with sediment filling
CGCM (2)	Dead bark	51	25	0.19	0.46	0.37	17 cobbles, no sediment filling
CGCM (3)	Dead bark	30	22	0.27	0.74	0.90	19 cobbles, looser packing of bark, with sediment filling
CGCM (4)	Dead bark	50	31	0.48	0.93	0.96	19 cobbles, with sediment filling
CGCM (5)	Dead bark	50	27	0.35	0.78	0.70	19 cobbles, with sediment filling
CGCM (6)	Dead Bark	50	25	0.30	0.72	0.60	19 cobbles, with sediment filling, max. temperature 259 °C
FGCM (1)	Dead bark	50	24	0.41	1.03	0.82	26 flint fragments, with sediment filling
FGCM (2)	Dead bark	50	18	0.09	0.28	0.18	26 flint fragments, with sediment filling

Three cobbles were used at the time for the condensation method.

The flint-groove condensation method followed a similar protocol as the cobble-groove condensation method. The construction of the flint-groove was also based on Todtenhaupt et al.’s^15^ descriptions. Because the flint pieces were smaller than the cobbles, we used six fragments to cover the bottom of the groove, five and six fragments on the wall sides, and one fragment at the bottom end which was lifted at times to allow better air circulation. After 50 g of birch bark strips were placed in the groove for both experiments, the structure was covered by eight additional fragments and the gaps between them were sealed with sediment. The operation of the experiments followed the same protocol as the cobble-groove condensation method, but the combustion of the bark only took 12 and 17 min.

## Results

All three methods, the condensation method, and the flint- and cobble-grooves produced birch tar. There was no wind when the experiments were conducted. In two of the eight groove experiments, we had to keep the fire burning by gently blowing air into the structure (one time). The scraping of the tar after each experiment took approximately 7 ± 1 min (we therefore added 7 min to the burning time of each run throughout the following text and in Table 1). Building of the groove structures is not counted in the times of Table 1 because they only had to be fully built once. We neglected the time necessary for bark refilling in Table 1, because it represented less than two minutes. In two experiments, the traditional condensation method produced a similar amount of tar after 30 min (0.27 g and 0.28 g), although the amount of bark used varied (Table 1). Hence, higher efficiency was achieved in the second session in terms of tar obtained per used bark. The first trial of the cobble-groove produced a relatively low amount of tar (0.16 g), but only about one-third of the bark was burned in this run. The second experiment, which was the only one where the gaps between the cobbles were not filled with sediment, produced 0.19 g of tar in 25 min (including the scraping). Using 30 g of bark in the third cobble-groove experiment resulted in a shorter run time (22 min). 0.27 g of tar was collected in this experiment (Fig. 2d). The fourth, fifth and sixth runs produced larger amounts of birch tar (0.48 g, 0.35 g and 0.30 g). The flint-groove condensation method produced 0.41 g of tar in the first experiment, but only 0.09 g in the second experiment. These represent the highest (experiment 1) and lowest efficiencies (experiment 2) of all experimental runs in this study. The temperature within the cobble-groove of experiment 6 fluctuated between 70 °C after lighting the bark and 259 °C after three minutes, which represents the maximum temperature recorded.

## Discussion

### Observations made during our experiments

In terms of efficiency, the cobble-groove proved to be more productive in experiments 3–5 after the structure was modified to include two additional cobbles that allowed increasing the depth of the structure. In a full hour, the groove could have produced close to 1 g of birch tar (the most efficient session producing 0.93 g/h), which is almost twice the amount obtained by the condensation method experiment (Table 1). We noted that in experiment 2 of the cobble-groove condensation method, where cobbles were not sealed with sediment, the tar yield was lower (0.19 g of tar in 25 min). The reason for this might be that some of the tar containing vapour was lost to the atmosphere, not resulting in tar condensation. This might be understood in the light of previous findings that tar is best formed in an anaerobic atmosphere^16^. We noted almost no difference of efficiency, as per tar/time and tar/bark, in the third run of the cobble-groove (using 30 g of bark) as compared to the other two runs with the same construction (using 50 g of bark in experiments 4 and 5). This suggest that the exact quantity of bark burned in a cobble-groove at a time is not of great importance. This hypothesis needs further testing in supplementary experimental runs performed with different amounts of bark. Another observation we made was that the 10° ground slope does not seem to be important for the method to work, as the 6th run of the cobble grove (with no ground slope) produced an amount of tar in accordance with the other runs (that were built with a 10° ground slope).

In addition to the higher efficiency achieved with the cobble-groove, the perceived effort needed is lower than for the condensation method. While the individual performing the condensation method needs to continuously pay attention to the burning of the bark rolls and scrape off the tar multiple times during the experiment, the only effort after lighting the bark in the groove consisted of blowing air and lifting the cobble placed at the lower end of the groove whenever the fire threatens to extinguish. This was necessary in two of the experiments.

As stated above, temperatures documented during one of the cobble-groove experiments (experiment 6) ranged between 70 and 259 °C. However, the temperature did not change gradually but fluctuated between these values. The maximum temperature of 259 °C was already reached three minutes into the experiment, which could refer to the moment that the flames reached the measuring tip of the thermocouple. The cobble-groove is an open structure allowing some airflow, and it can be expected that the temperatures are not uniform at different locations within the structure. Due to these fluctuations and the fact that we did not measure the temperature at different locations within the structure, we do not think that our temperature measurement allows us to make comparisons between the temperature of the cobble-groove and other birch tar making techniques.

During the first experiment using the flint-groove, we observed the highest efficiency. This may be due to the smooth surface of flint in comparison to the rougher surface of the cobbles. Consequently, tar would not be lost within the irregularities of the rocks. Similarly, it is possible that the surface irregularities of our cobbles filled up with tar during the first two experiments, leading to a higher tar yield during the later three runs. The second flint-groove experiment, on the other hand, did not yield a large amount of tar. After only 12 min (excluding the scraping), the flames went extinct and upon removal of the sediment and cover rocks, we noted that the bark strips had only partially burned (Fig. S6m of the supplementary material). This was the only instance that the fire went out completely. When scraping the tar off the flint fragments after the first experiment, we struggled to hold the flint in our hands as the rocks had become hot. By the end of the second experiment, some flint fragments were broken (see for example Fig. S6j of the supplementary information file). Flint is prone to internal fracturing when excessively heated^24^, and hence we argue that the same flint fragments could not have been used for a third tar making session. Another question is whether the flint flakes used resemble intentionally heat-treated flint artefacts. Heat-treated flint cannot be recognised macroscopically if it is not knapped after the heat treatment (see for ex:^22^. It is therefore unlikely that flint flakes resulting from the flint-grove condensation method for making birch tar would be taken for intentionally heat-treated rocks. The reaction kinetics and resilience to heat propagation in flint (see for ex:^25^ make it unlikely that the burning time of a single flint-groove condensation method run would produce sufficient transformations in the flint to improve its knapping quality. They are rather expected to lead to overheating (compare:^24^).

### Comparison of our method with other known birch tar making techniques

Within the ongoing debate about birch tar making techniques that is mainly fuelled by experimental research, only the more recent publications provide detailed descriptions of the parameters and results that allow us to extract data on efficiency. Recent findings by Kozowyk et al.^9^ describe the efficiency of three allothermic methods—ash mound, pit roll, raised structure—providing details on the parameters used as well as the results obtained. The methods, ranging from simple (ash mound) to complex (raised structure) result in varying tar yields^9^. The ash mound consists of a bark roll that is covered with ash and embers. The highest yield collected from within the bark layers was 1 g per 100 g of bark^9^. For the pit roll method, a birch bark roll is placed within a small pit with a recipient (e. g. pebble) at its bottom to collect the tar. Hot embers are placed on top of the bark roll. The most successful run produced 2.4 g per 100 g of bark^9^. The raised structure, an earth mound structure build over a bark roll placed on a mesh, produced 15.7 g in one attempt^9^. This refers to 9.6 g per 100 g of bark. The raised structure was replicated in a more recent study^7^ and, in the most efficient experiment described therein, produced 22.7 g of tar per 100 g of bark. Todtenhaupt et al.^15^ produced 1cm^3^ (~ 1.14 g) per 100 g of bark, which is more than double the efficiency of our replication of Schmidt et al.’s^14,22^ condensation method (0.54 g per 100 g of bark). Our replication using the autothermic cobble-groove condensation method produced 0.96 g per 100 g of bark, which is still almost double the amount of tar obtained by the conventional condensation method.

It is not straightforward to directly compare these methods in terms of time efficiency. The condensation method is the only method that can be performed for a potentially unlimited amount of time. Although the cobbles need to cool down between production sessions, this could be avoided by exchanging the cobbles for each run. With the condensation method, it is possible to actively produce birch tar during, e.g. 30 min. This is not possible for the pit roll or ash mound methods, as the burning time cannot be shortened or prolonged (in other words, the time is dictated by the progression of the experiment). The cobble-groove is more efficient in terms of tar/h (maximum values would be 0.93 g/h for the cobble-groove and 1.03 g/h for the flint-groove) as compared to the condensation method (~ 0.5 g/h). Although the groove structure requires an initial set up time of about 12 min, the overall time requirement decreases in successive runs because the structure only needs to be refilled and covered again. Comparing the condensation method with the raised structure is equally difficult because the raised structure requires to be operated for 3–4 h and this is not adjustable. The raised structure produces the highest tar yield, but it cannot produce tar in a short time, such as the two condensation methods. The limitations of the flint-groove condensation method include the internal fracturing of the fragments upon exposing them to excessive heat and longer cooling intervals between the tar making sessions.

Schmidt et al.^14,22^ argue that although complex techniques for birch tar making cannot be ruled out, the null hypothesis must be that the simplest method was used for making the known Neanderthal tar pieces. Although birch tar making is often associated with anaerobic conditions, e. g. in raised structures, our successful trials with the cobble-groove and flint-groove prove that birch tar can be produced by several techniques that operate in aerobic conditions. We also note the resemblance of the residue-covered cobbles discovered at Inden-Altdorf with the cobbles left behind by the experiments conducted for this study^6^.

### Comparison of our method with archaeological tar

The results of our study can also be regarded in comparison to the birch tar artefacts recovered at Neanderthal sites. This comparison allows to appreciate the potential time requirement for producing these artefacts with the cobble-groove. Time efficiency may be an important factor if, for example, tar was quickly needed for a specific task or if large amounts were required.

The smaller birch tar artefact discovered at Königsaue in Germany weighs 0.87 g and the larger piece weighs 1.38 g^2,3^. It is unclear if these values refer to the original weight of the tar pieces, as the degradation of organic substances may have resulted in weight loss. Thus, these following numbers must be seen as minimum values. As an example, it is possible to produce 0.87 g of tar in 93 min using the condensation method and, at least theoretically, in 56 min using the cobble-groove (if generalised from the most successful experiments CM 2 and CGCM 4). The FGCM (based on experiment 1) could have produced this amount in 50 min, if the flint fragments did not fracture or were exchanged with unheated fragments. The Zandmotor tar piece has a volume of 1.990 mm^3^^5^. Admitting a density of 1.14 g/ml for wood tar^26^, the approximate weight of the Zandmotor piece is 2.27 g. The time needed to produce this amount with the condensation method would be ~ 4 h while the cobble-groove could produce it in ~ 2 h 30. Although it is possible to calculate a production time of ~ 2 h 15 with the flint-groove, this would require multiple new sets of flint fragments. Again, we might be able to perform the condensation method for a long time period if we exchange the cobbles between the sessions. But the cobble-groove would need to be modified to burn for a longer time, e. g. by making it longer so that it can contain more bark. The raised structure, for example, can produce significantly greater tar yield in general but it is not possible to produce the amount of tar known from archaeological contexts in less than 245 min [i.e. the shortest time span determined experimentally by Kozowyk et al.^9^ including fire preparation and set up]. Unfortunately, no measurements of size or volume are provided for the Campitello tar and considering the unknown volume of the stone tool hidden underneath, we cannot make a precise estimation of volume or weight.

### Implications of the material used in our study

The materials used in our study, such as the cobbles used for the condensation method and the cobbles and flakes used for the groove structures, can be seen as potential artefacts left behind in the archaeological record. The patterns of tar residues, even after the scraping, are still clearly recognisable on the surface that was exposed to the flames (for examples see Fig. S4 for the cobble-groove stones and Fig. S6 for the flint flakes in the supplementary information). Tar stains remained on the wall and roof cobbles/flint flakes of the groove experiments. No tar was collected on the stones that built the bottom. Therefore, these cannot be recognised as indicators of tar production. The top and wall stones show similar tar patterns and could not be distinguished after the experiment in terms of their position in the structure. Single finds of cobbles, such as the Inden-Altdorf cobble (if confirmed to be birch tar), might be comparable to the cobbles used in the condensation method. Evidence for use of the cobble-groove, however, would require the discovery of multiple tar-covered cobbles. Still, the cobbles might have been reused after the tar production, or only the tar-covered stones removed, leaving behind the paved bottom stones. These considerations are of importance as future finds of cobbles or flint flakes covered in tar could be understood to point towards birch tar production.

During our experiments, we used small, sharp flint flakes to scrape the tar off the cobbles and the flint fragments. These flakes are an easily accessible and producible material that could have been used by Neanderthals to retrieve the tar off their production stones. After the experiments, tar residues were still visible on these flakes (Fig. S6k and l of the supplementary material), which, if comparable artefacts should be found in the future, might also allow us to make comparisons between artefacts and our experimental material.

## Conclusion

In this study, we pursued the aim of replicating two variations of the condensation method that might have been employed by Neanderthals in the Middle Palaeolithic and which could have served to produce tar pieces such as those known from Campitello^1^, Königsaue^2–4^, and Zandmotor^5^. Both methods, the condensation method proposed by Schmidt et al.^14,22^ and the cobble-groove condensation method modified after the method described by Todtenhaupt et al.^15^, proved to be successful in producing birch tar in each experimental run. Although we do not provide any data on how Neanderthals produced birch tar, we note that the cobble-groove method could have been employed to obtain the amount of tar known from archaeological contexts.

In terms of experimental archaeology, future directions could include experimentation with different types of cobbles, ranging from rough to very smooth surfaces and exploration of birch tar making with the same cobbles over an extended period to evaluate whether the thickening of tar on the cobble over time influences the condensation of new tar. We also expect that modifying the size of the cobble-groove (e.g. longer or higher) could further increase the tar yield without increasing the complexity of the experiment. While the experimental making of birch tar cannot prove the exact procedure employed by Neanderthals in the Middle Palaeolithic, it can provide a range of examples of how it might have been possible. Only further findings of tar as well as the material used for its production could provide adequate proof of how Neanderthals might have produced tar and give insight into potential development and improvement of the techniques employed.

## Supplementary Information

Supplementary Information 1.

Supplementary Video 1.
