# Supplementary material for: A new method for birch tar making with materials available in the Stone Age
Source: Sci Rep. 2022 Jan 10;12:413. doi: 10.1038/s41598-021-04161-3 (PMC8748610; doi:10.1038/s41598-021-04161-3)
Supplement: Supplementary file 1 — Supplementary Information 1. [file 41598_2021_4161_MOESM1_ESM.pdf]

A new method for birch tar making with materials available in the Stone Age

Tabea J. Koch[1] & Patrick Schmidt[1, 2]

[1] Department of Early Prehistory and Quaternary Ecology, Eberhard Karls University of Tübingen, Germany.
[2] Applied Mineralogy, Department of Geosciences, Eberhard Karls University of Tübingen, Germany.

Corresponding author:
Tabea J. Koch (Orcid: 0000-0001-9907-2554)
Mail: tabeakoch@palaeome.org
Patrick Schmidt (Orcid: 0000-0002-8727-6127)

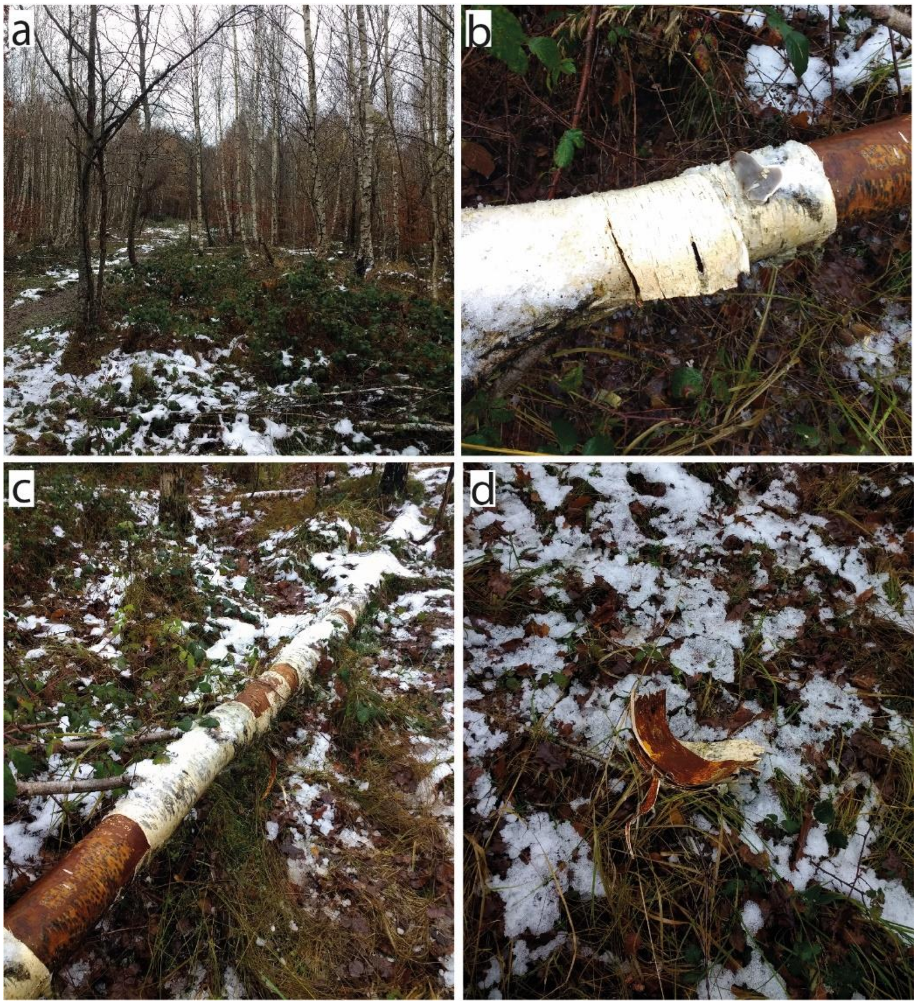

Supplementary figure 1. Birch bark collection for the groove condensation methods. (a) Birch forest. (b) Cutting of the bark with a sharp flint flake. (c) Areas of well-preserved bark that were collected. (d) Bark strips cut off the birch trees.

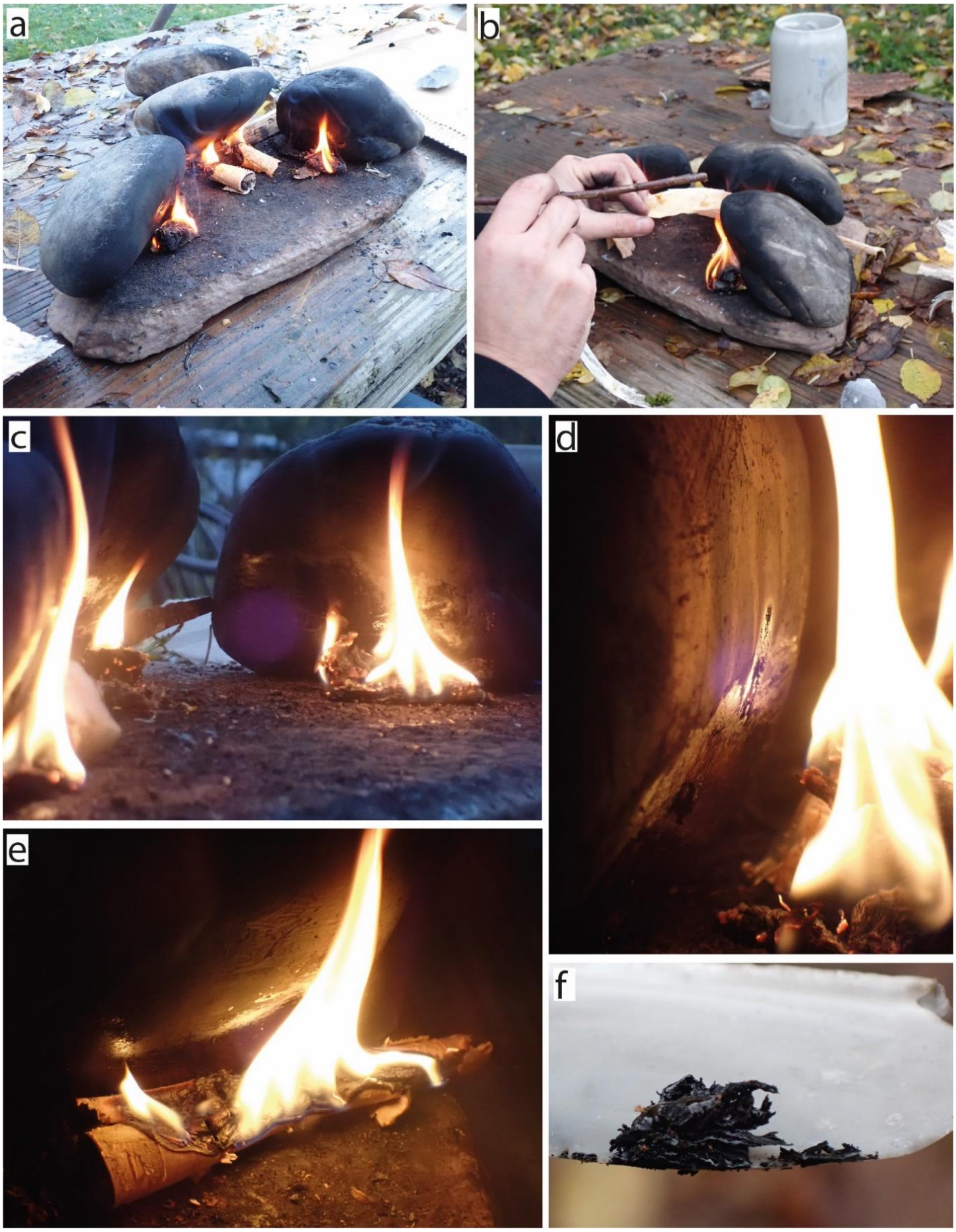

Supplementary figure 2. Experimental setup of the birch tar making experiment with the condensation method. (a) Overview. (b) Lighting of bark during the experiment. (c-e) Close-up images of the tar condensation onto the flat cobble surfaces. (f) Tar scaped off with a flint flake.

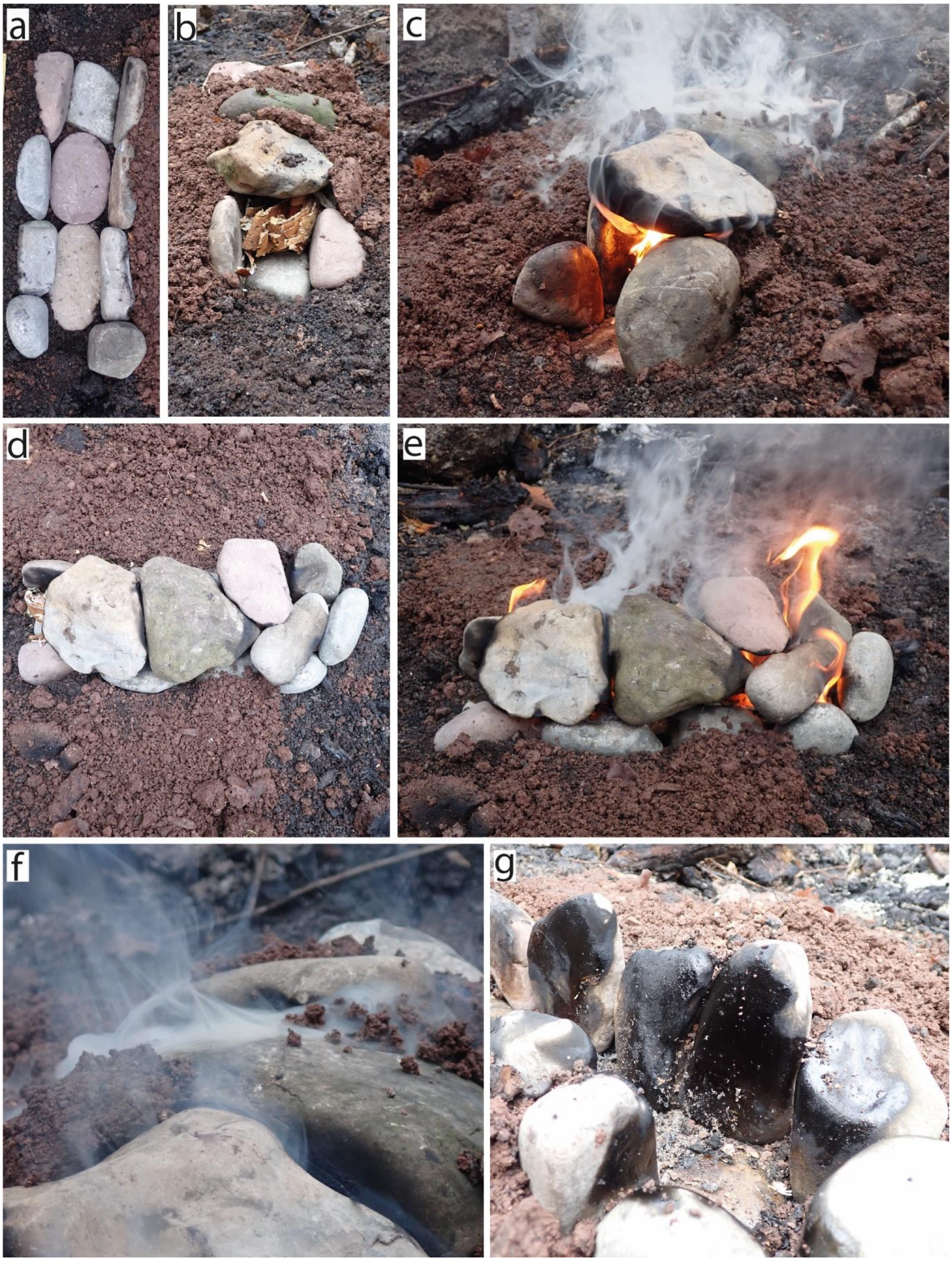

Supplementary figure 3. Experimental setup of the birch tar making experiments with the cobble-groove condensation method. (a) The groove before the first run; (b) the open end of the structure covered with cover stones and gaps infilled with sediment; (c) experiment while running. (d-e) Second experimental run, in which the spaces between the cover cobbles were not filled with sediment. (f) Close-up image of the smoke exiting the structure during the fifth run. (g) Wall side cobbles before scraping of the tar.

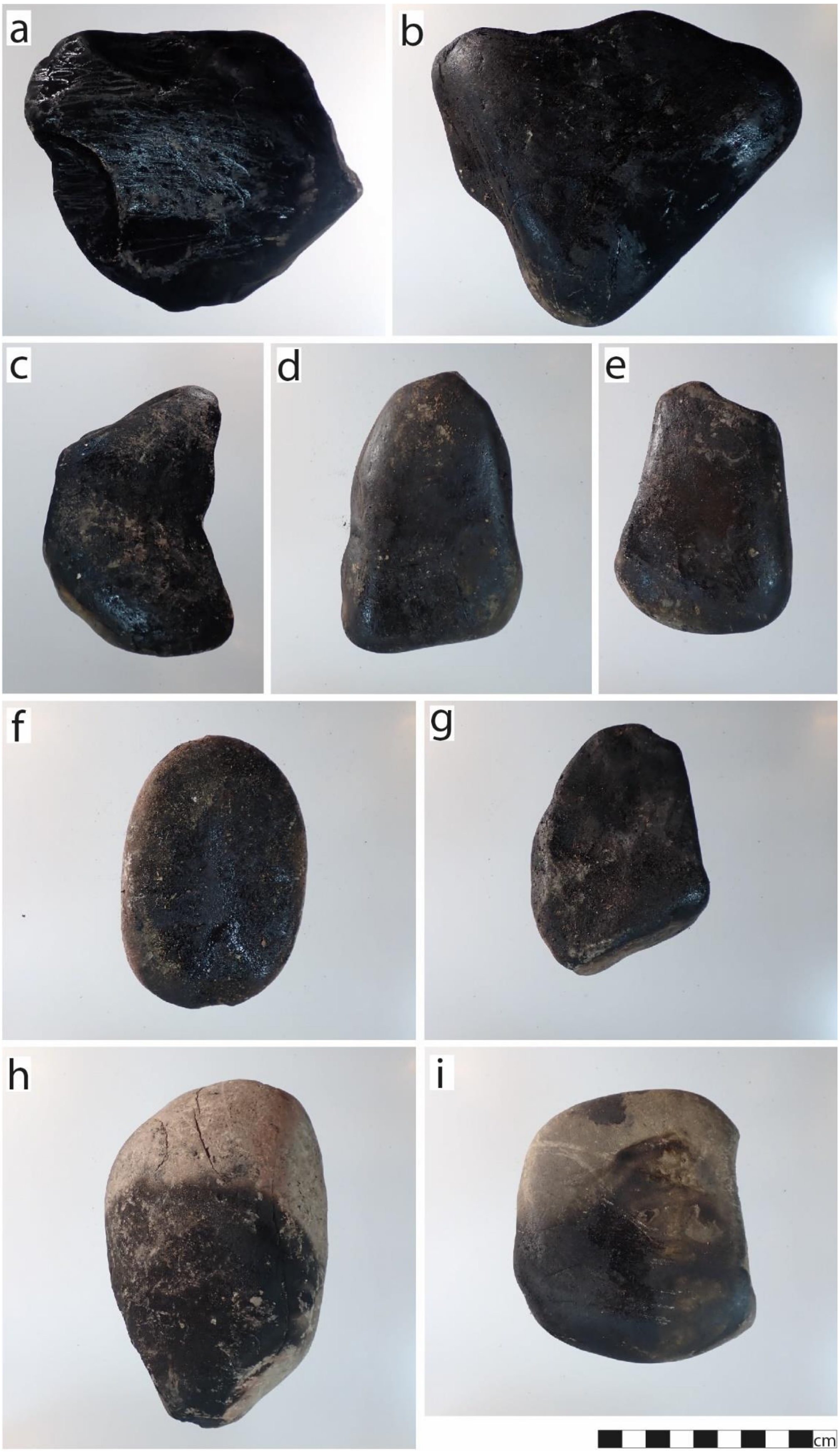

Supplementary figure 4. (a-i) Selection of the cobbles used in the cobble-groove experiments. The images show the cobbles after the five runs and scraping of the tar.

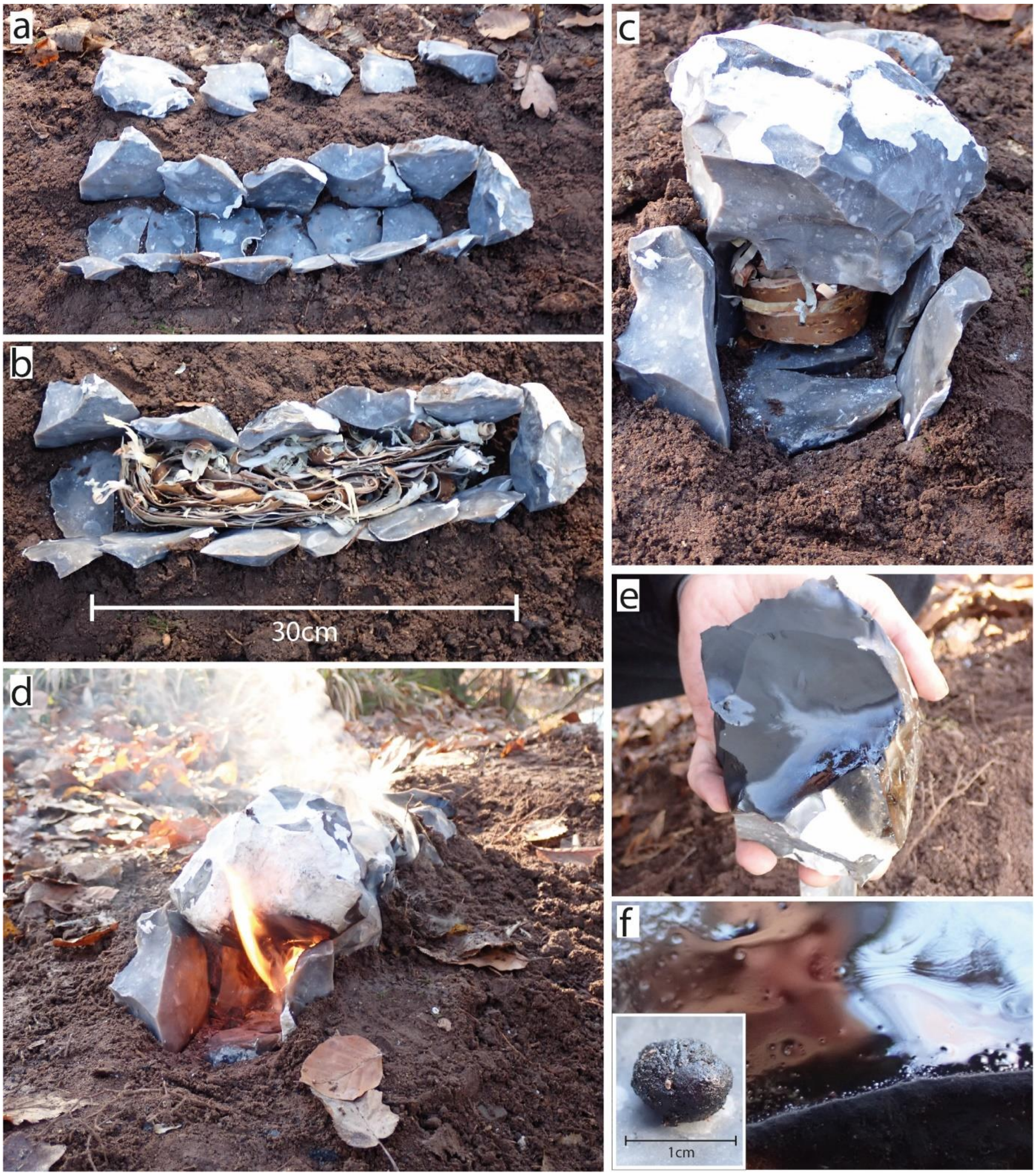

Supplementary figure 5. Experimental setup of the birch tar making experiment with the flint-groove condensation method. (a) The groove structure before the first run; (b) flint-groove filled with 50g of birch bark strips; (c) open end of the structure where the bark is to be lit and after lighting (d). (e) flint piece covered in tar before the scraping; (f) close-up photo of condensed tar on a flint and a 0.41g weighing piece of tar produced in 17 minutes.

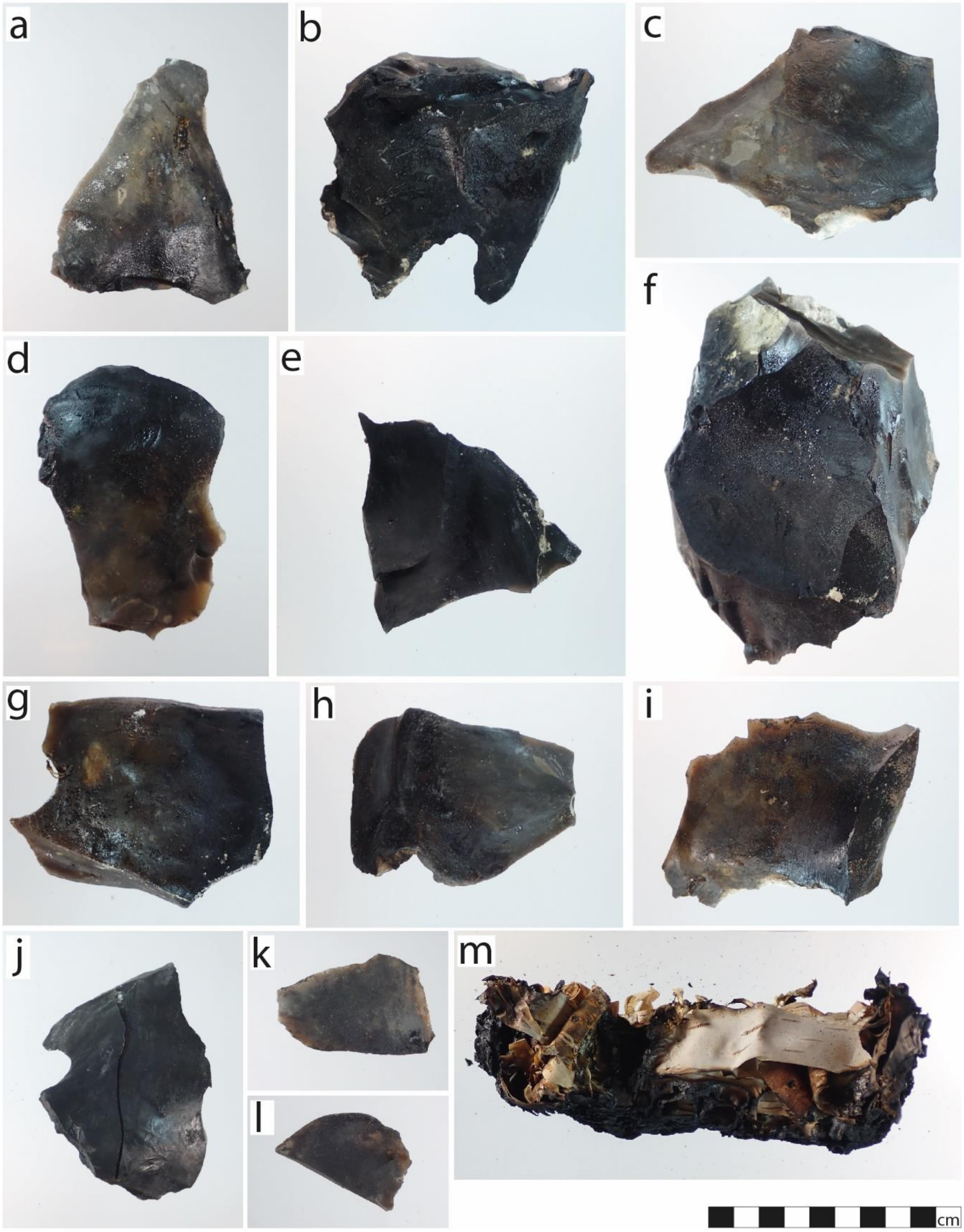

Supplementary figure 6. (a-i) Some of the flint pieces used in the flint-groove experiments. The images show the pieces after the two runs and scraping of the tar; (j) flint piece that broke during the second experimental run; (k-l) flint flakes that were used for scraping tar, showing residues on the cutting edge. (m) Partially burned birch bark strips that were left in the second experimental run.
